# Real-time driver monitoring system with facial landmark-based eye closure detection and head pose recognition

**DOI:** 10.1038/s41598-023-44955-1

**Published:** 2023-10-25

**Authors:** Dohun Kim, Hyukjin Park, Tonghyun Kim, Wonjong Kim, Joonki Paik

**Affiliations:** 1 Electronics and Telecommunications Research Institute, 22, Daewangpangyo-ro 712beon-gil, Bundang-gu, Seongnam-si, Gyeonggi-do Republic of Korea; 2 Department of Image, Chung-Ang University, 84 Heukseok-ro, Seoul, 06974 Korea; 3 TQS Korea, 406ho, B, Jiphyeonjungang 7-ro, Sejong-si, Korea; 4 CANLAB, 604ho, Daewootechnopia 296, Sandan-ro, Danwon-gu, Ansan-si, Gyeonggi-do Korea; 5 Department of Artificial Intelligence, Chung-Ang University, 84 Heukseok-ro, Seoul, 06974 Korea

**Keywords:** Electrical and electronic engineering, Computer science, Information technology, Software

## Abstract

This paper introduces a real-time Driver Monitoring System (DMS) designed to monitor driver behavior while driving, employing facial landmark estimation-based behavior recognition. The system utilizes an infrared (IR) camera to capture and analyze video data. Through facial landmark estimation, crucial information about the driver’s head posture and eye area is extracted from the detected facial region, obtained via face detection. The proposed method consists of two distinct modules, each focused on recognizing specific behaviors. The first module employs head pose analysis to detect instances of inattention. By monitoring the driver’s head movements along the horizontal and vertical axes, this module assesses the driver’s attention level. The second module implements an eye-closure recognition filter to identify instances of drowsiness. Depending on the continuity of eye closures, the system categorizes them as either occasional drowsiness or sustained drowsiness. The advantages of the proposed method lie in its efficiency and real-time capabilities, as it solely relies on IR camera video for computation and analysis. To assess its performance, the system underwent evaluation using IR-Datasets, demonstrating its effectiveness in monitoring and recognizing driver behavior accurately. The presented real-time Driver Monitoring System with facial landmark-based behavior recognition offers a practical and robust approach to enhance driver safety and alertness during their journeys.

## Introduction

With the rapid advancement of computer vision technology, various AI applications related to vehicles, including autonomous driving, have become subjects of extensive research. However, in the realm of mobility, vehicle accidents resulting from driver drowsiness, alcohol consumption, and negligence in maintaining front-view attention continue to be prevalent each year. To address these concerns, regulatory agencies worldwide are proactively responding to technological advancements in the automotive industry. The European Commission, for instance, has issued recommendations for driver monitoring technology and regulations to enhance vehicle safety ratings^1^. Starting in 2022, the European Commission regulations will mandate the incorporation of driver monitoring technology. Similarly, the US National Transportation Safety Board (NTSB) recommends the adoption of Driver Monitoring Systems (DMS) in semi-autonomous vehicles^2^. Despite the availability of commercially accessible autonomous driving technology at levels 2.5 to 3, which require driver attention to avoid potential accidents due to user carelessness, the need for robust DMS remains paramount. DMS is designed to analyze the driver’s condition and detect potentially hazardous situations, such as drowsy driving, drunk driving, and lapses in front-view attention. As the risks associated with traffic accidents escalate based on the driver’s abnormal state, the significance of implementing DMS systems continues to grow.

Various techniques have been explored to analyze the driver’s state, including drowsiness and inattention, as evidenced by previous studies^3–5^. Traditional Driver Monitoring Systems (DMS) typically assess driver behavior based on driving patterns, like drift-and-jerk, which were developed before the era of deep learning technology and autonomous driving functions^6^. These systems utilize the OBD-II protocol to collect driving data, and driver status is analyzed by integrating the driving patterns with a computer vision system^7^. Researchers have also explored the use of computer vision technology to analyze the driver’s state based on facial features. However, RGB cameras face challenges in real-world applications due to environmental variations such as changing lighting conditions and complex backgrounds. To overcome these limitations, some studies have adopted IR cameras to parameterize facial features for computer vision techniques^8,9^. Analyzing the driver’s eye features and gestures plays a crucial role in assessing their state. Various studies have examined the driver’s eye information and gestures to understand their condition^10–12^. Moreover, with the advent of deep learning, there has been notable research on driver status analysis through behavior recognition using Convolutional Neural Network (CNN) networks^13,14^. Various methodologies exist for driver state analysis systems. Among them, image classification-based methods are prominent for discerning a driver’s facial expressions and overall state. Recently, the vision transformer technique has gained attraction in the field of computer vision^15^. Moreover, to ensure reliability and manage multi-modal data, decision fusion-based image classification techniques can be employed^16^. Consequently, many recent driver monitoring systems have adopted deep learning technologies. However, when embedding deep learning-based computer vision into systems, further research might be needed to counter adversarial attacks^17–19^.

In this paper, we present a DMS that utilizes images captured with an IR camera to detect driver drowsiness and inattention. To identify these states based on video footage alone, it is necessary to analyze the driver’s behavior. In the proposed method, we begin by detecting the driver’s face, after which we extract facial landmarks for two primary purposes: head pose estimation to identify inattentive situations and eye closure recognition to detect drowsy driving. Head pose estimation enables analysis of the driver’s gaze direction, while the newly proposed eye-closure recognition filter is used to determine whether the driver is drowsy or not. This paper presents an extension of Jung’s method^20^. The focus of this work is to provide a comprehensive description of the proposed system, outlining the functionality of each component in detail. To facilitate the collection of video data for real-world product development, we developed an in-vehicle video transmission device. The performance evaluation of our proposed method was conducted using a custom-made dataset called “irdatasets” (Supplementary Information)^21^. The data set used in this study was self-recorded by the author and is included in this published article for analysis. Informed consent was obtained from all subjects for publication of image in the online open access publication

## Can-lab DMS camera

To create a robust deep learning-based Driver State Monitoring (DSM) system capable of handling lighting variations caused by the rotation of proposed IR LEDs, we conducted video analysis using a camera developed in-house by Can-lab^22^. Table 1 presents the DSM camera’s performance under the assumption of OEM vehicle installation.

**Table 1:** DSM camera’s performance under the assumption of OEM vehicle installation.

Number of output pixel	1280 × 800
Dynamic range	63.9 dB
Signal to noise ratio (SNR)	MAX 39 dB
Frame rate	30 FPS
Power supply voltage ratings	DC 12V ± 0.5V
Transmission interface	GMSL2
Operating temperature range	− 40 + 85 °C
Storage temperature range	− 40 + 125 °C

**Figure 1:**
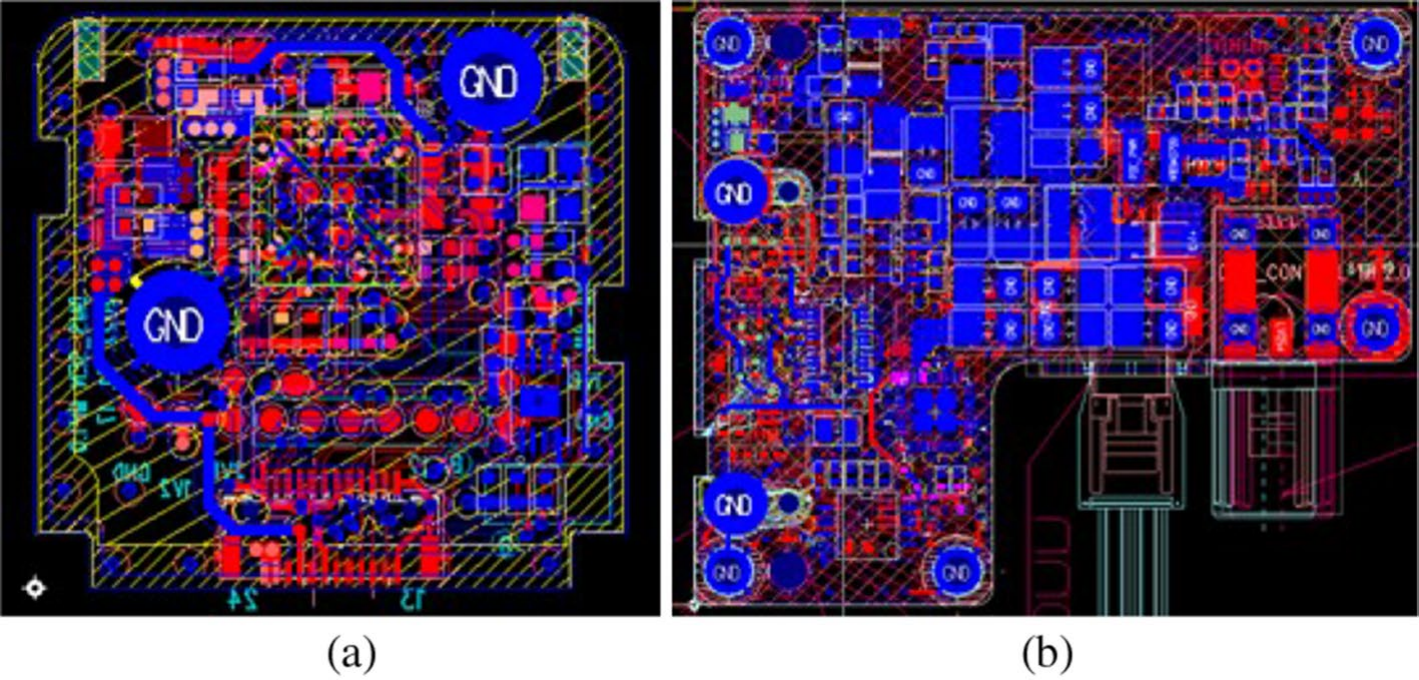
Artworks of the DMS camera: (**a**) Image sensor and (**b**) control board.

Our camera design incorporates essential features such as IR LED control functionality and seamless communication between the camera and the Electronic Control Unit (ECU). Additionally, to enhance video quality by reducing noise, we employed a separate power supply for the IR-LED Driver. These considerations were vital in optimizing the DSM system’s effectiveness and performance.

The camera’s Image Sensor and control board artwork are illustrated in Fig. 1. Figure 1a showcases the image sensor artwork, while Fig. 1b displays the control board artwork. To ensure efficient heat management, the prototype of the DMS Camera employed a strategically dispersed arrangement of high-temperature components in the circuit design. Furthermore, we prioritized the use of automotive-grade materials to enhance the camera’s overall durability and reliability. The camera’s optimal IR Printed Circuit Board (PCB) was fabricated by conducting temperature testing based on the PCB area, as detailed in the paper. This meticulous approach contributed to the camera’s robust performance and thermal stability.

**Figure 2:**
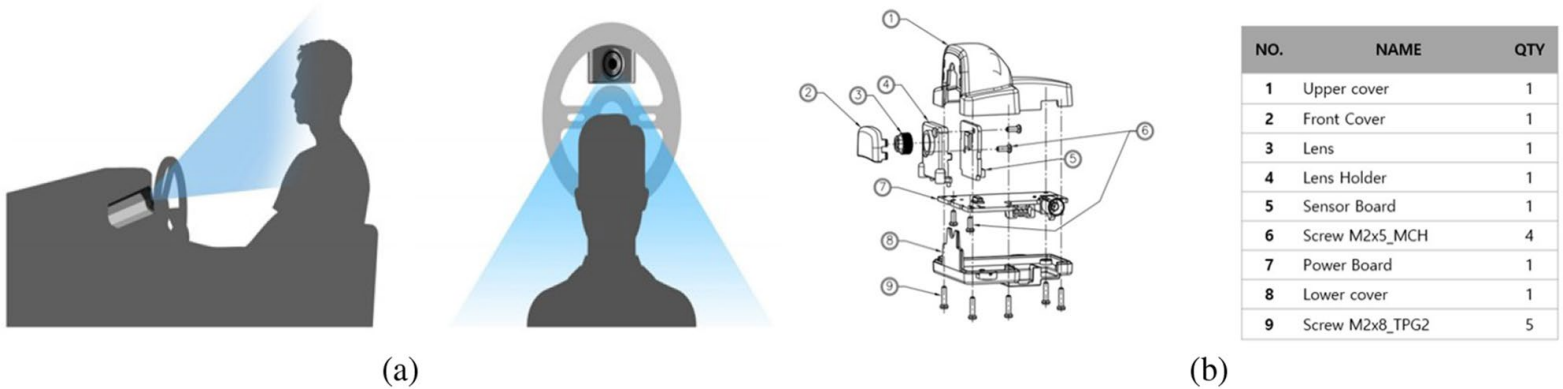
Configuration of the Driver Monitoring System: (**a**) Installation example and (**b**) internal compnent configuration.

In the proposed Driver Monitoring System (DMS) described in this paper, a custom-made image acquisition device developed by CANlab is utilized for capturing images. The configuration of this image acquisition device is depicted in Fig. 2, where Fig. 2a shows the mechanical assembly of the camera, and Fig. 2b displays the positioning of the DMS camera inside the vehicle.

**Figure 3:**
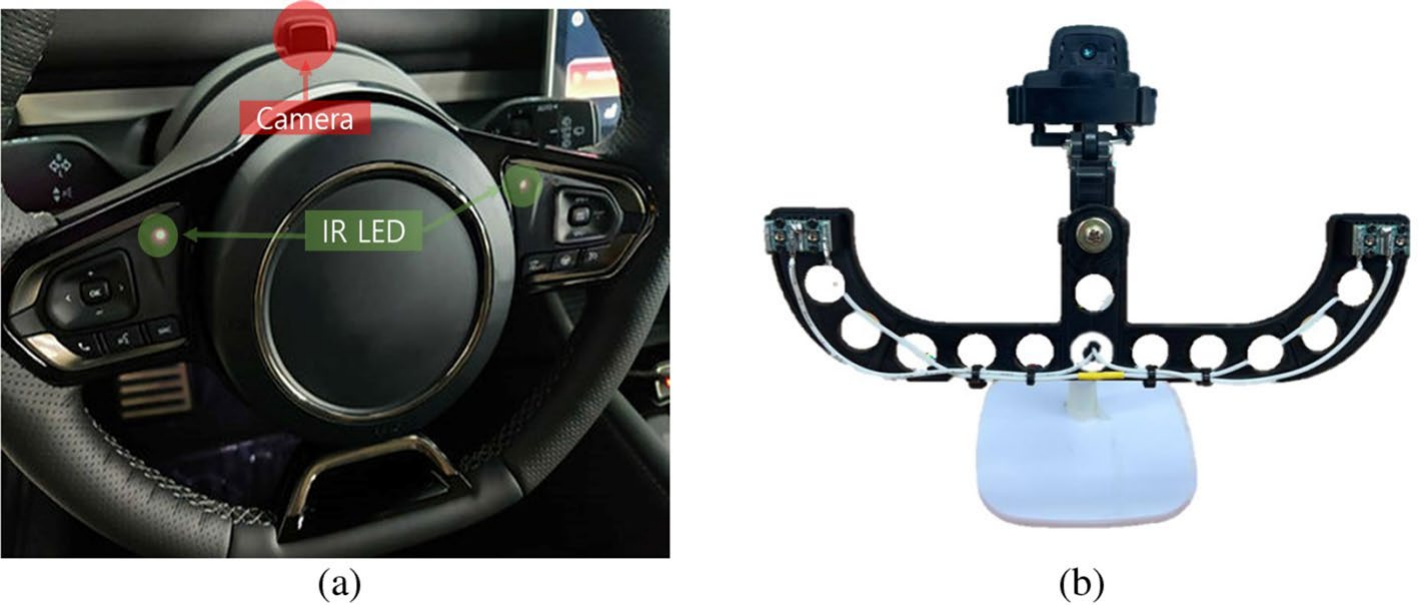
DMS camera in CANLAB: (**a**) DMS camera mounted on a vehicle and (**b**) prototype of the DMS camera.

**Figure 4:**
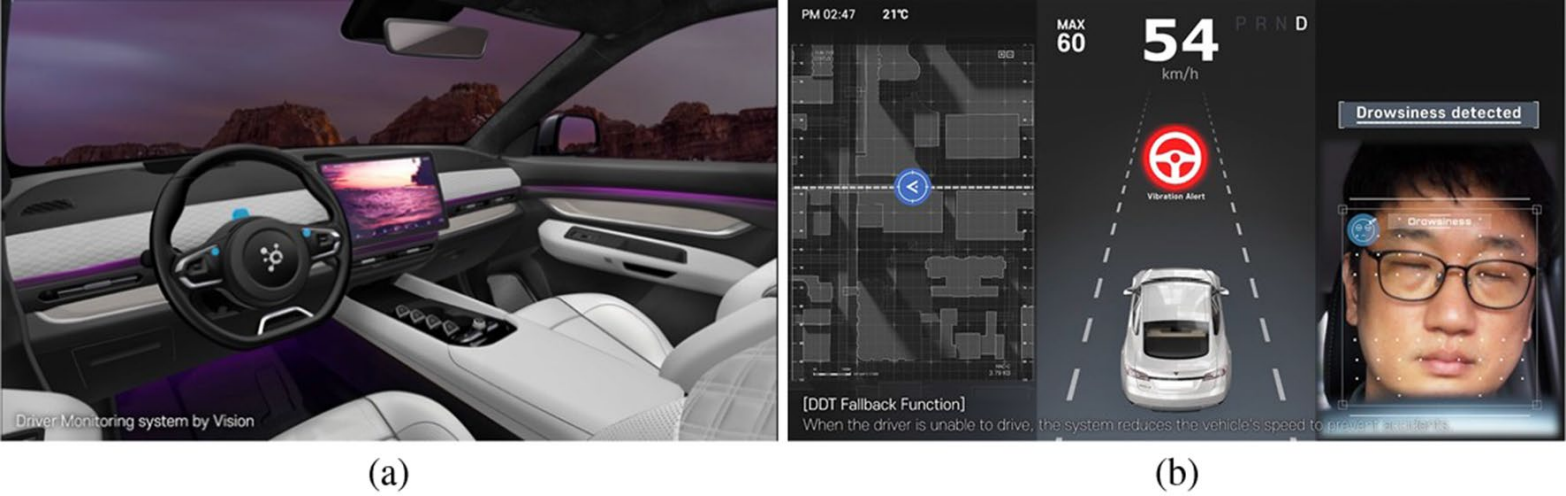
Prototype view of the proposed DMS: (**a**) The DMS camera installed at the center of the steering wheel and (**b**) sample images demonstrating the operation of the DMS.

This paper presents a system that employs a camera, crafted by CANLAB, to capture and subsequently analyze the driver’s condition using the obtained images. Figure 3 illustrates the genuine installation of the proposed DMS camera alongside its prototype images intended for testing. The camera finds its place on the steering wheel column. Figure 3a displays the authentic installation while Fig. 3b offers a view of the test prototype. Thus, its strategic placement behind the fixed steering column ensures that it remains undisturbed during any steering wheel adjustments.nd exploration to pave the way for its market entry. Figure 4a provides an anticipated prototype view of the proposed DMS, illustrating its installation at the center of the steering wheel for optimized analysis of the driver’s face. Figure 4b showcases sample images demonstrating the operation of the DMS using the aforementioned camera. These images display the driver state analysis results, obtained by continuously matching information from the steering wheel with the driver’s current state. In the following sections, a comprehensive explanation will be provided regarding the methodology employed for driver state analysis, as depicted in Fig. 4b.

## Feature extraction for driver status analysis

**Figure 5:**
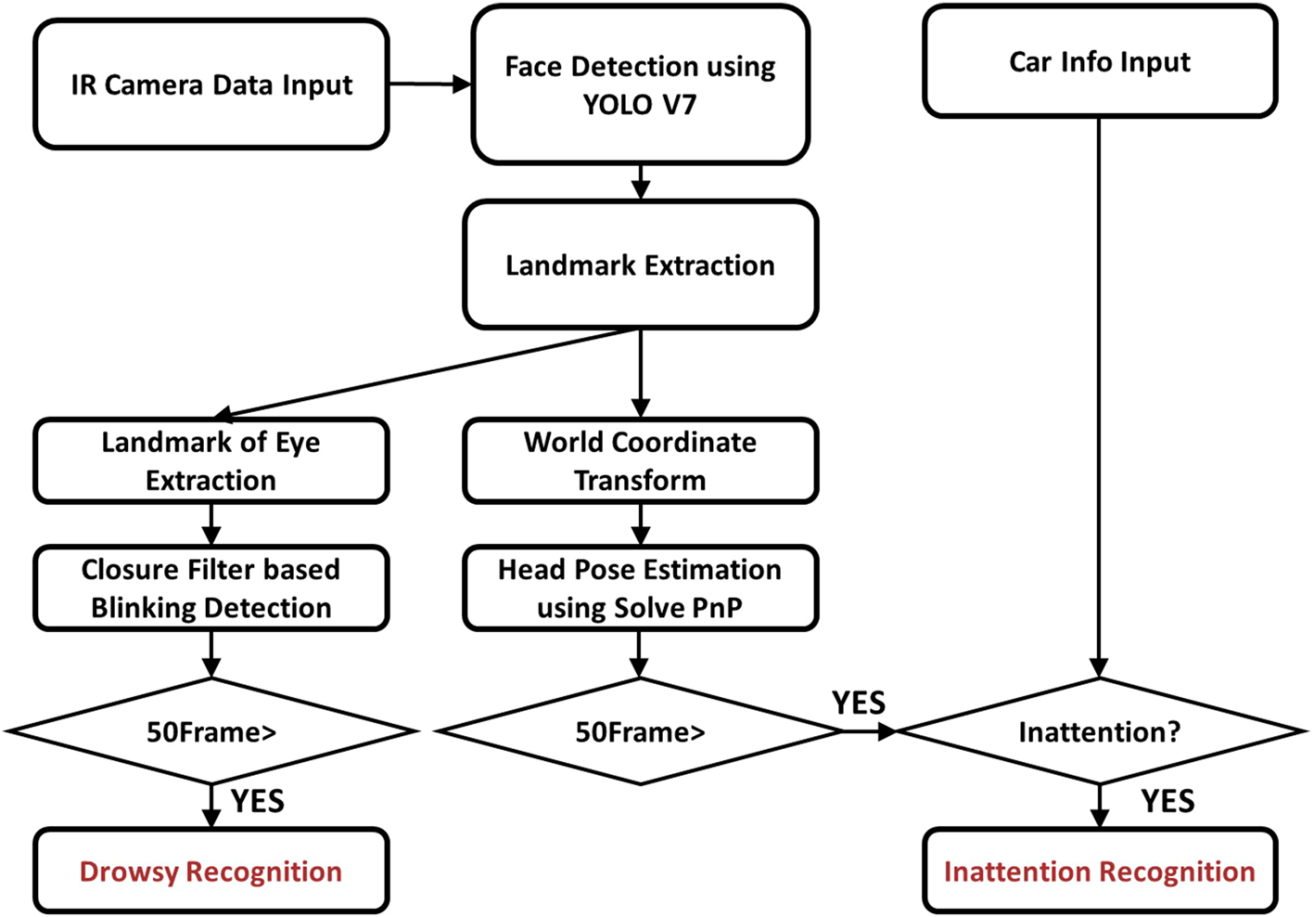
Flow chart of the proposed method.

In this section, we will delve into the extraction of facial features that enable an analysis of the driver’s state. To detect instances of drowsy and inattentive driving, we have designed a Driver Monitoring System (DMS), as depicted in Fig. 5. The DMS employs an IR camera as the input image source, and the captured image is used for facial landmark estimation. These extracted facial landmarks serve as crucial features for estimating head pose and identifying instances of closed eyes, which are indicative of drowsy driving.

In our proposed system, facial detection algorithms play a crucial role as a preliminary step for estimating eye blinking and head pose, which are essential for driver state analysis. The images captured in the system have a resolution of 1280 × 800, and Fig.4b illustrates that the driver’s face is consistently positioned at the center of the image. Due to the controlled environment for image collection, unaffected by rotation, background complexity, lighting conditions, and varying object sizes, several facial detection algorithms, such as SSD^23^, Faster RCNN^24^, and Efficient Det^25^, have shown excellent performance. After evaluating various high-performing algorithms, we opted to utilize the YOLOv7^26^ network for our system. YOLOv7 is a 1-stage detection algorithm, similar to the SSD detector, offering both satisfactory performance and fast processing speed. For training the YOLO detector, we employed custom datasets along with wild datasets. By extracting facial features based on the detected faces in the images, we enable the detailed analysis of the driver’s state.

**Figure 6:**
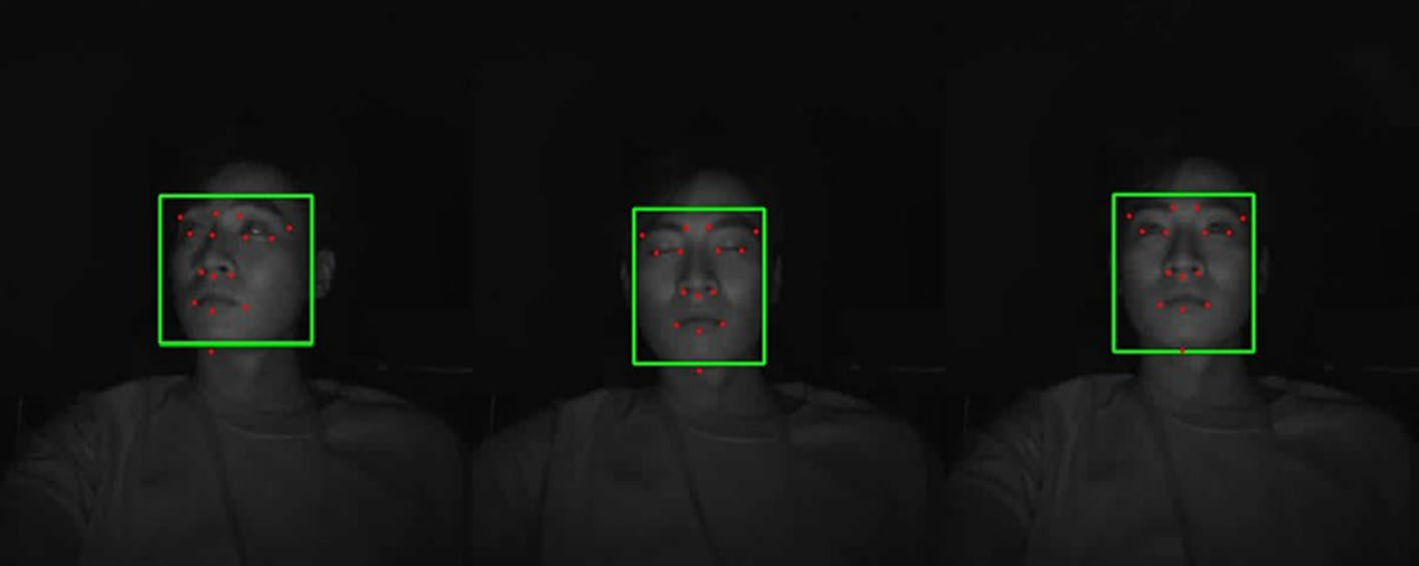
Face detection result with estimated facial landmark points in IR Images.

Facial landmark detection has witnessed significant advancements in recent years, with deep learning techniques like Openface^27^ and Retinaface^28^ leading the way. However, in the proposed Driver Monitoring System (DMS), there are considerable costs associated with face detection using deep learning and eye closure detection through image filters. To address this, we have opted to employ Kazemi et al.’s fast facial landmark extraction algorithm^29^. Kazemi’s algorithm is based on random forests and offers a favorable combination of fast execution speed and good performance. Considering that the DMS needs to operate in an embedded environment rather than a desktop environment, the random forest-based method is well-suited for the proposed system. An experimental video showcasing face landmark detection using IR Images is provided in Fig. 6. These extracted landmarks serve as essential features for driver state analysis, and comprehensive explanations of their use will be provided in the following section.

## Face feature extraction for driver status analysis

The proposed driver state analysis algorithm focuses on recognizing two major risk factors of driving accidents: (i) drowsy driving and (ii) inattentive driving behavior. While conventional Driver Monitoring Systems (DMS) typically integrate software analysis information like eye closure, nodding, and forward gaze detection with hardware analysis information such as steering data and vehicle speed, this paper does not address the latter due to limited access to a vehicle’s ECU data, which is controlled by the manufacturer. Instead, our paper proposes a system that leverages a single camera for analyzing the driver’s state. In the proposed DMS system, we can still extract hardware information, such as the steering angle of the vehicle’s handle and whether the vehicle is in motion or parked, and integrate it with the proposed software. By incorporating the logic of vehicle control information into the proposed software, we can create a comprehensive DMS system. This section outlines how we analyze drowsiness and distracted driving behavior solely based on data captured by a single camera. By focusing on these critical factors, our system aims to enhance driving safety and reduce the risk of accidents caused by driver fatigue and inattention.

### Inattention analysis method

To identify driver inattention, our system initiates the head pose estimation process. Head pose estimation provides crucial information about the driver’s head angle and gaze direction, which is essential for recognizing head gestures and determining forward gaze. We look for signs of drowsy driving, such as shaking the head from side to side or consistent nodding, as well as indications of the driver’s focus on the road ahead based on the orientation of the head when the steering wheel is fixed.

Head pose estimation can be categorized into two main approaches: (i) deep learning-based head pose estimation algorithms and (ii) solvePnP estimation-based head pose estimation algorithms. While deep learning-based methods have the advantage of not requiring separate face landmark extraction^30,31^, they have limitations when it comes to classifying learned classes and may not provide numerical analysis for each frame, which can lead to errors in practical applications. Moreover, using resource-intensive algorithms in real-time systems, which demand processing data in real-time, can lead to frame drops.

**Figure 7:**
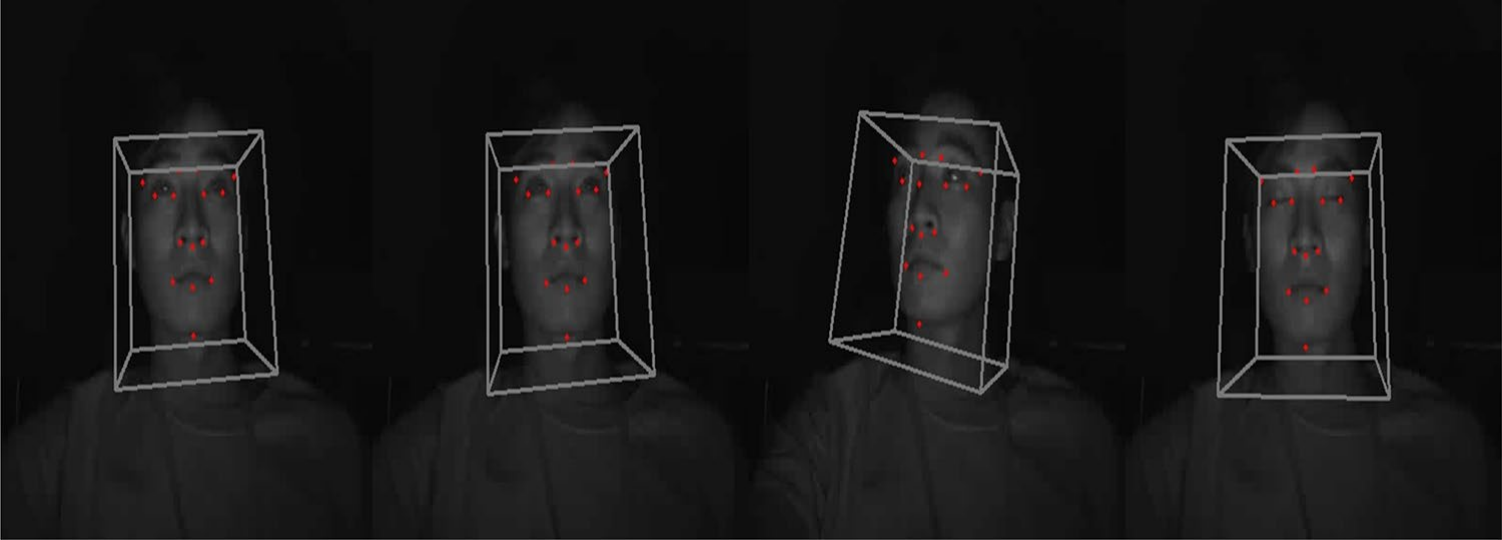
Head pose estimation results from IR Images.

In this paper, we opt for a head pose estimation method based on the solvePnP estimation algorithm of face landmarks^32^. The PnP-based head pose estimation algorithm determines head pose by establishing the correspondence between the 2D coordinates and 3D coordinates of the extracted face landmarks. The 3D coordinates we want to estimate exist in the world coordinate system, and with knowledge of the translation vector and rotation vector, we can project the corresponding 2D coordinates onto the 3D coordinate system. In our work, we estimate head pose using the solvePnP algorithm provided by OpenCV^32^. The solvePnP algorithm estimates rotation and translation vectors and converts 2D coordinates to 3D coordinates using DLT (Direct Linear Transform) and Levenberg–Marquardt optimization^33,34^. Figure 7 illustrates the head pose extracted using the solvePnP algorithm.

We propose an algorithm that utilizes the angular information extracted from this head pose estimation to determine the orientation of the head and subsequently analyze the forward gaze situation. By employing this approach, we aim to accurately assess the driver’s attention to the road, providing valuable insights for ensuring safe driving conditions.

### Drowsiness analysis method

To effectively analyze drowsy driving, it is crucial to identify instances when the driver closes their eyes. In our proposed method, we employ an eye-closing detection filter to recognize such situations. Existing object detection algorithms’ approach to detecting eye-closing and eye-opening instances proves challenging due to frequent occurrences of false positives and false negatives.

**Figure 8:**
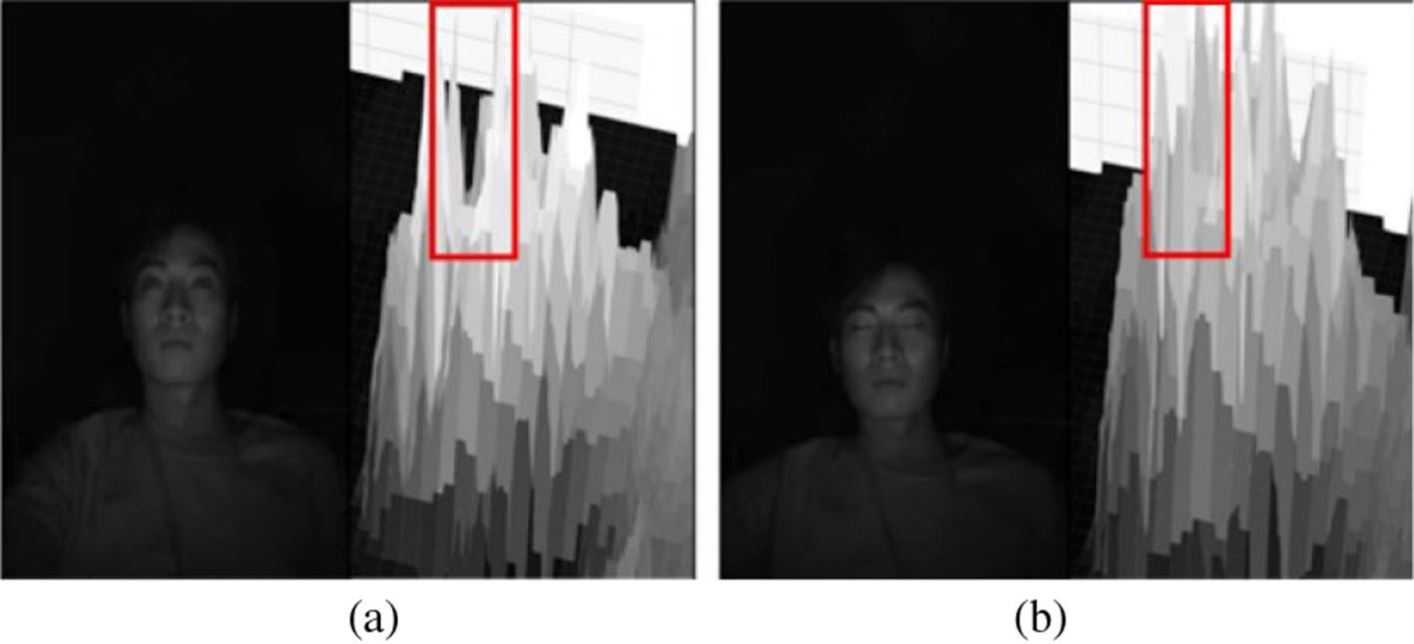
Visualization of depth results in the IR Image: (**a**) Normal and (**b**) drowsing.

In our method, we address this challenge by determining the optimal threshold value to accurately analyze the eye-closing area in the image. Figure 8 provides a visualization of the depth in the infrared (IR) image based on pixel values. This visualization aids in understanding how the proposed method discerns eye-closing situations.

By fine-tuning the threshold value and employing the eye-closing detection filter, our system strives to reliably detect when the driver closes their eyes, contributing to an improved assessment of drowsy driving behavior and enhancing overall driving safety.

Figure 8a demonstrates the visualization result when the driver’s eyes are open. As the pixels corresponding to the eyes have brightness values close to 0, it is evident that blank spaces appear in the visualization. Conversely, Fig. 8b shows the result when the driver’s eyes are closed, with the eye area being filled with bright pixels.

To discern between open and closed eye states, our proposed method utilizes a threshold value based on the brightness values of the pixels. The process begins by extracting the cropped eye image using facial landmarks. Subsequently, we calculate the threshold value *T* using Eq. (1), which averages the minimum and maximum pixel values in the image *I*. This threshold value is then employed for binarization, where pixels greater than *T* are set to white, and pixels less than or equal to *T* are set to black.

1 $$\begin{aligned} {T=\frac{min(I)+max(I)}{2}} \end{aligned}$$

**Figure 9:**
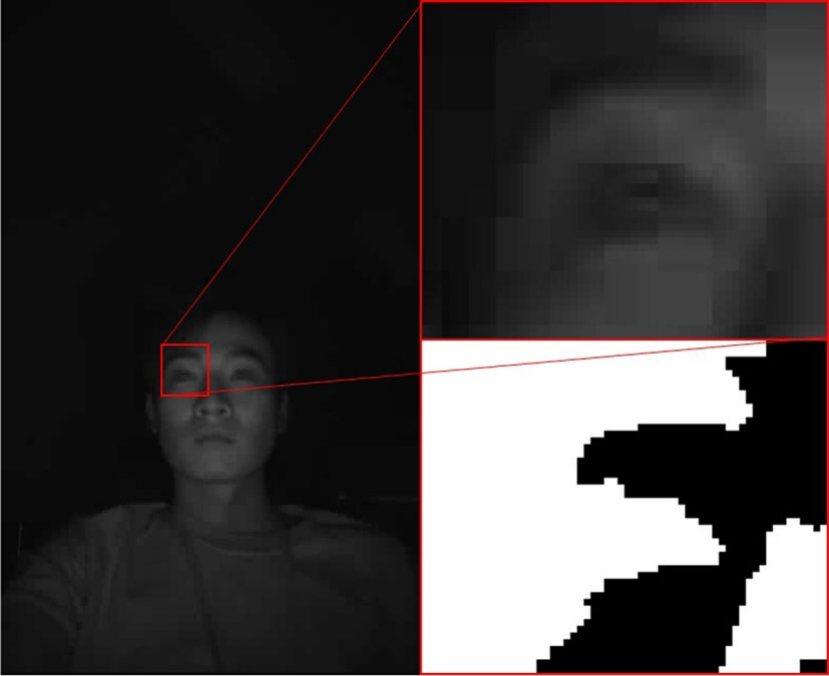
Result of thresholding process on the eye region.

The resulting binarized image, obtained through this thresholding process, is depicted in Fig. 9. By effectively applying this binarization technique, our method accurately identifies the eye state, facilitating reliable detection of eye-closing instances. This contributes to a robust analysis of drowsy driving behavior, ultimately enhancing the overall safety of the driving experience.

**Figure 10:**
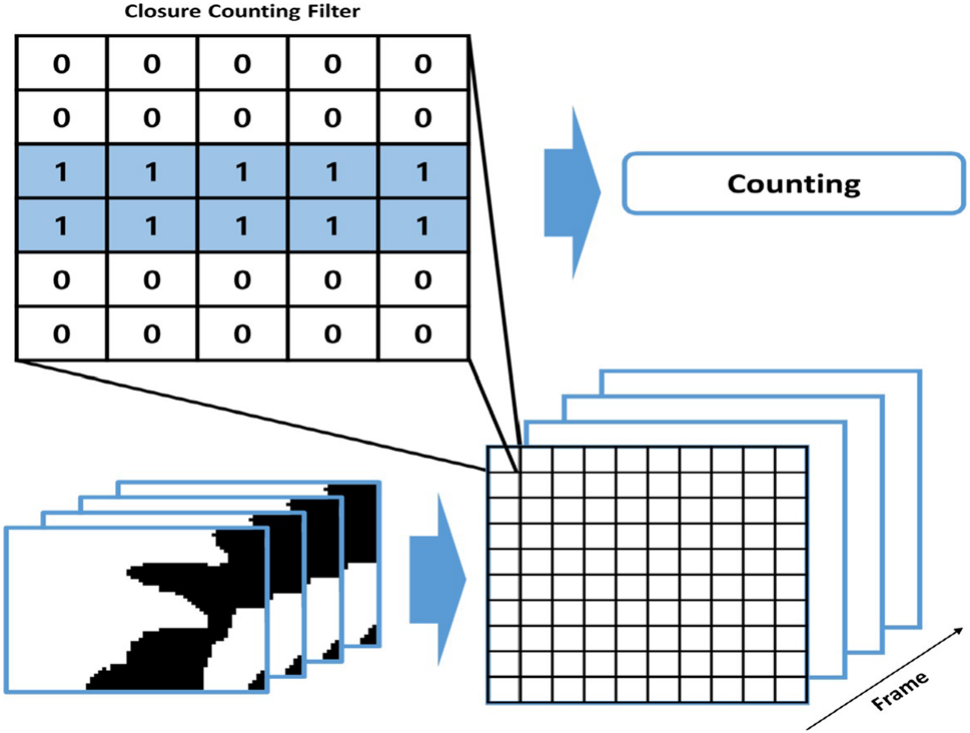
Eye closure recognition filter for drowsiness recognition in IR Images.

By performing threshold processing, as shown in Fig. 9, the low pixel values of the eyes enable clear identification of eye features. To detect these characteristics, we apply an eye-closure detection filter based on the image filter depicted in Fig. 10. This proposed filter scans the image by sliding over it and searches for pixel areas that meet specific conditions. When the filter is applied to situations where the eyes are open, no satisfying area is found. However, when the filter is applied to situations where the eyes are closed, as demonstrated in Fig. 8b, the closed features of the eyes are accurately detected.

Our proposed method proves suitable for detecting drowsiness situations as it can precisely identify the eye area compared to deep learning-based algorithms and offers fast processing speed. We define a drowsy situation when the number of pixels detected through the filter exceeds 40 and persists for 50 frames or more.

This approach provides an effective means of detecting drowsy driving instances by analyzing the eyes’ behavior. By reliably identifying closed-eye situations using the proposed filter, we can enhance driver safety by timely alerting or intervening in drowsy driving scenarios, ultimately preventing potential accidents.

## Experimental results

In this section, we conduct a comprehensive performance evaluation of the proposed method using the IR driver state analysis dataset. The dataset comprises two types of situations: normal and drowsy. It was acquired using an IR camera and includes instances under low-light conditions. The dataset’s configuration is presented in Table 2.

**Table 2:** Configuration of IR datasets.

Events	Image	Events	Image
Normal (40)		Normal (low-light) (40)	
Drowsy (40)		Drowsy (low-light) (40)	
Common condition			
Camera: IR Camera			
Distance: 30–50 cm			
Resolution: 1280 × 800			
Video file: AVI file encoded			
Video length: within 80–120 frame			

The camera is positioned in front of the driver’s seat, capturing individuals at a distance of 30-50cm. The recorded video is in AVI format with a resolution of 1280 × 800, and each file typically consists of 80-120 frames. Before evaluating drowsiness and inattentiveness detection in drowsy drivers, we first assessed the face detection performance under both low-light and normal lighting conditions. The YOLO V7 model used for training was trained on separate data from the test data, and its performance is summarized in Table 3.

**Table 3:** Experimental results of face detection in IR Images.

Contents	Frame	Detected frame	Confidence (%)	Precision (%)	Recall (%)
Normal	22,379	22,027	92.4	100	98.4
Low-light	17,996	17,993	98.2	100	99

The confidence value in the table represents the average confidence of the detected faces. We achieved a precision of 100%, indicating that no false detections were observed in either low-light or normal lighting conditions. For Recall values, which denote the percentage of correctly detected faces, we obtained 98.4% and 99% for low-light and normal lighting conditions, respectively. These values correspond to a minor percentage of missed frames, approximately 2%, indicating that the majority of frames were accurately detected.

Having successfully evaluated the face detection performance, we now proceed with the performance evaluation of the proposed method using the IR dataset to analyze drowsiness and inattentiveness detection in drivers.

2 $$\begin{aligned}{} & {} {Precision=\frac{True \, Positive}{True \, Positive+False \, Positive}} \end{aligned}$$

3 $$\begin{aligned}{} & {} {Recall=2\times \frac{True\, Positive}{True\, Positive+False \, Negative}} \end{aligned}$$

### Drowsiness recognition

In this subsection, we present the performance evaluation of the drowsiness detection algorithm utilizing an eye-closure recognition filter. As previously explained, recognizing eye closure is a critical step in detecting drowsy situations. To achieve this, we leverage the Adaptive threshold feature in the IR camera, which allows us to differentiate areas based on brightness values.

**Figure 11:**
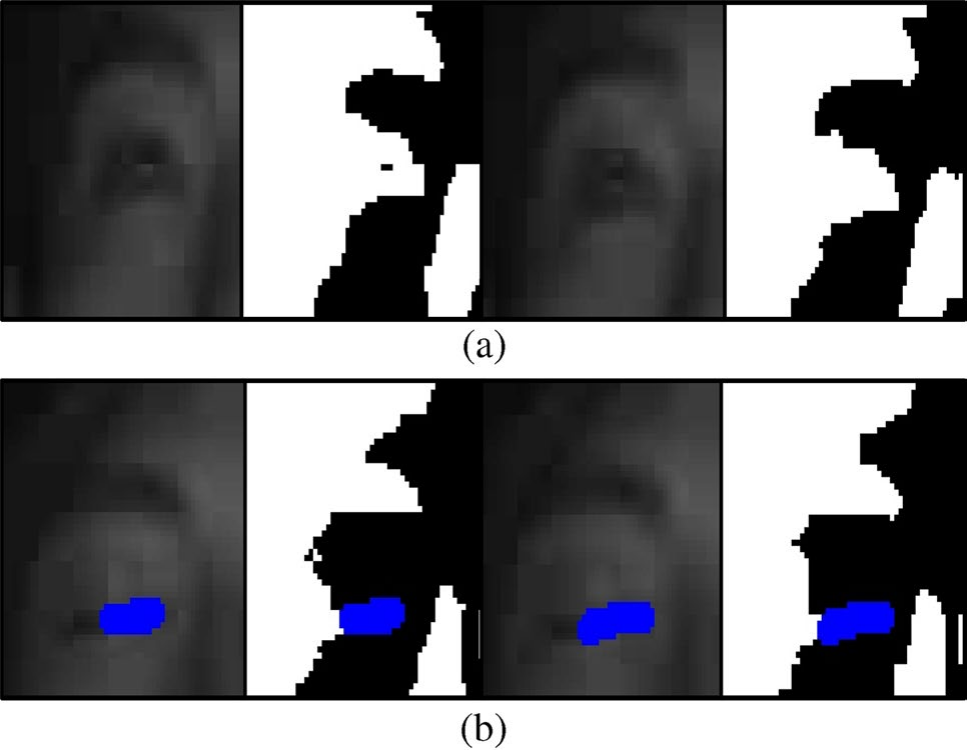
Experimental results of eye-closing detection using eye-closing recognition filter.

In our proposed method, we count the pixels obtained using the eye-closure filter from the image generated through threshold processing. The result obtained using the eye-closure filter is illustrated in Fig. 11. In Fig.11a, which represents a normal situation, no matching points are found in the area, thus characteristic values cannot be obtained. However, in Fig. 11b, the filter successfully identifies the characteristic values for the area where the eyes are closed.

To determine a drowsy situation, our proposed method sets a threshold of 40 or more points, lasting for 50 frames or more. If these conditions are met, the situation is recognized as drowsy. The experimental results for recognizing drowsiness behavior are summarized in Table 4. Both the accuracy and precision achieved are above 99%, indicating a highly accurate detection of drowsy driving.

**Table 4:** Experiment results for drowsiness detection using IR dataset.

Contents	Normal	Normal (low-light)	Drowsy	Drowsy (low-light)
Count	40	40	35	35
Accuracy	100%			
Precision	99.3%			

With these results, we demonstrate the effectiveness of our eye-closure recognition filter in accurately identifying drowsy driving behavior, contributing to improved driver safety and accident prevention.

### Inattention recognition

**Figure 12:**
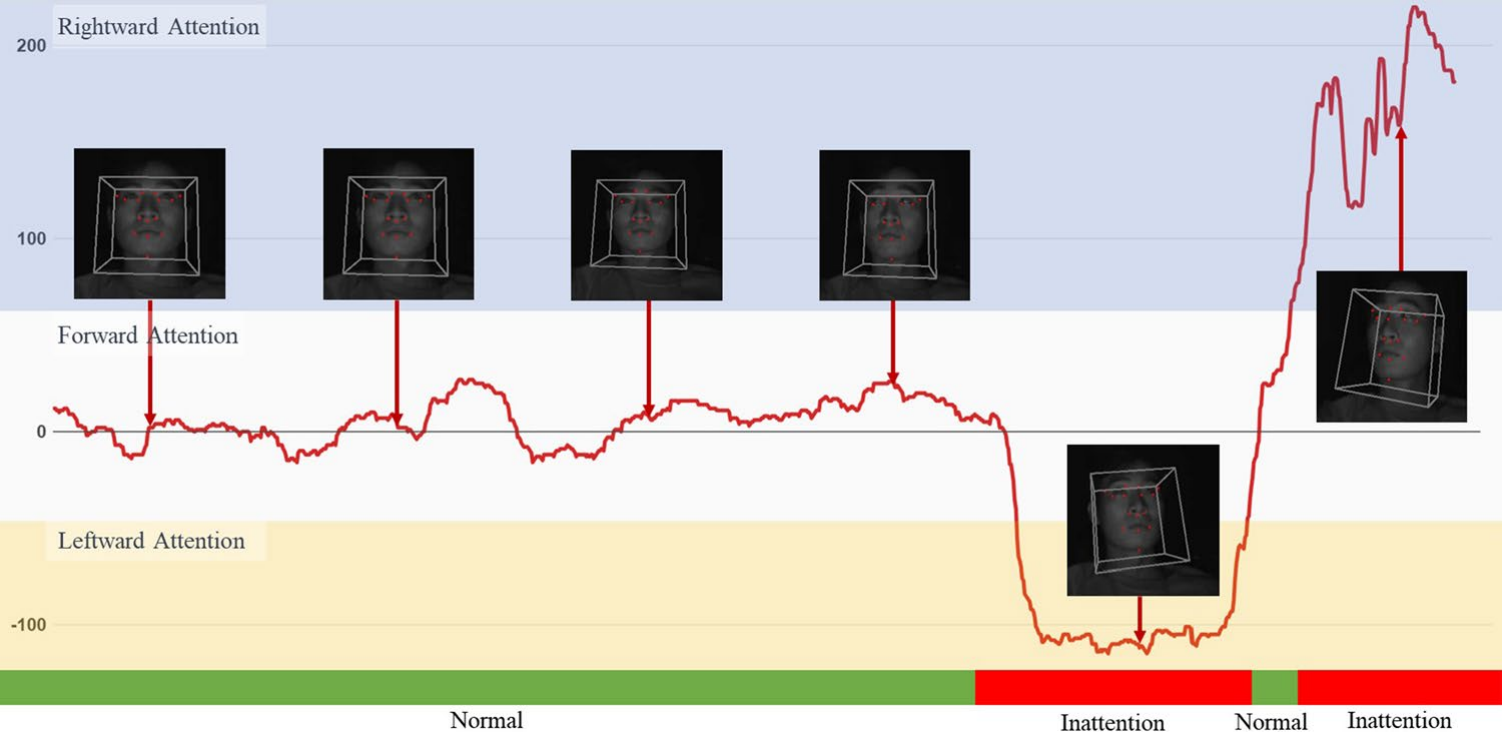
Experimental results of head pose estimation.

The inattention analysis method utilizes the head pose obtained in Section “Feature extraction for driver status analysis” for analysis. Through the estimated head pose, we can determine the driver’s head direction. In the proposed method, we record the driver’s head movement along the *x* and *y* axes to monitor the forward-looking situation. If the head direction does not face forward for more than 50 frames, it is recognized as an inattentive situation. Figure 12 shows a chart that represents head movements. As depicted, a rightward gaze results in an upward trajectory on the y-axis, while a leftward glance causes a descent along the negative axis. The red zone beneath the image denotes instances of inattention, whereas the green segment stands for regular situations. The graph’s blue portion pertains to moments when the gaze shifts right, and the yellow section marks a gaze directed left. By utilizing these graph patterns, we can identify inattentive behavior based on the direction of the head. Unlike deep learning-based methods, the algorithm used in this study allows for real-time analysis of head pose estimation results, reducing the risk of misrecognition.

In this paper, we analyze inattentive situations based on a single image. The experiment with the test dataset yielded a performance of over 99% for inattentive situation recognition, similar to the results obtained for drowsy driving detection. It is worth noting that the analysis was solely based on software information as there was no access to vehicle ECU data. In the future, more precise recognition of inattentive situations can be achieved by integrating hardware information. By accurately analyzing the driver’s head movement and detecting inattentive behavior, our proposed method enhances the overall safety of driving. The combination of drowsy driving detection and inattention analysis contributes to a comprehensive Driver Monitoring System (DMS) that provides valuable insights to prevent potential accidents and ensure a safer driving experience.

In this experiment, we conducted research on face recognition, drowsiness detection, and inattentiveness analysis using an IR camera. The IR camera demonstrated excellent performance in both low-light and normal lighting conditions, with little discernible difference between the two situations even to the naked eye. For drowsiness detection, we employed an eye-closure recognition filter instead of a learning-based method, and the results displayed an impressive detection accuracy of over 99%. Regarding inattentiveness analysis, while we could not perform exhaustive experiments due to the lack of integration with vehicle sensors, the test results indicated a success rate of over 99% in estimating the direction of the driver’s face. The processing speed of the proposed system was remarkable, achieving 20–25 frames per second (FPS) in the CPU I7-12700 with 32GB RAM and RTX 3060 environment. Even when utilizing the embedded board Xavier with Tiny YOLO, the processing speed remained at 10 FPS. This performance demonstrates the feasibility of applying the proposed method to real-world vehicles for commercialization. Overall, our experimental findings indicate the robustness and effectiveness of the proposed method for face recognition, drowsiness detection, and inattentiveness analysis. With the potential to enhance driving safety and prevent accidents, the proposed system holds promise for practical implementation in commercial vehicles.

**Table 5:** A review of existing DMS methods and sensor utilization.

Author	Primary methodologies	DMS type	Datasets	Modalities used
Du et al.^35^	1D-CNN	Direct	UTA-RLDD^36^	Visual RGB
Huang et al.^37^	MTCNN, LSTM		NTHU-DDD^38^	
Vijay et al.^39^	CNN, XGBoost			
Ahmed et al.^40^	MTCNN, InceptionV3			
Chen et al.^41^	MTCNN, ONET		Custom datasets	Frontal visual RGB, oblique visual RGB
Bakker et al.^42^	MTCNN, CE-CLM, AU-CNN, DLT-LM			IN-visual RGB, out-visual RGB
Lollett et al.^43^	GRU-based			Visual RGB
Sharak et al.^44^	RFC, XGB			Visual RGB, NIR, thermal
Ansari et al.^45^	LSTM			Motion capture
Tran et al.^46^	CNN, VGG, ResNet			Two visual RGB
Awais et al.^47^	SVM			Four visual RGB, ECG
Lin et al.^48^	SVR, MLPNN, RBFNN, Five-layer SONFIN			EOG, ECG, EEG
Massoz et al^49^	PDM, CLM, RLMS			ECG, EOG, EEG, EMG, Visual RGB, NIR, depth map
Kiashari et al^50^	ORD			NIR, thermal
Quddus et al^51^	LSTM			EEG, two NIR
Du et al^52^	MPF	Hybrid		Visual RGB, vehicle telemetry, audio, driving simulator
Kundinger et al^53^	SMOTE			Visual RGB, ECG, pulse, physioloigca, driving simulator
Baccour et al^54^	SVM			Visual RGB, driving simulator
Longxi Luo^55^	DSN			Visual RGB, driving simulator
Ours	YOLO v7, dlib	Direct	Custom datasets	IR

**Table 6:** Performance of DMS type^54^.

	Indirect DMS		Indirect DMS		Indirect DMS	
Awake	84.3%	15.7%	89.6%	10.4%	90.5%	9.5%
Drowsy	28.6%	71.4%	15.4%	84.6%	15.1%	84.9%

### Comparative experiment with existing driver monitoring systems

In the previous section, we described the experimental results of the DMS proposed in this paper. In this section, we aim to provide a review of the proposed system in comparison to existing DSM systems. Detecting driver drowsiness is a pivotal component of driver state analysis systems. Table 5 presents a compilation of previous DMS research endeavors focused on monitoring drowsy driving. These studies have employed various methods, datasets, and modalities to construct DMS. The “DMS Type” refers to the classification introduced by Baccour et al., which encompasses direct, indirect, and hybrid models. Direct methods involve analyzing the driver’s own characteristics, such as their face and eyes, for monitoring. Indirect methods, on the other hand, analyze factors like steering wheel angle information and vehicle vibrations to assess driver state. Hybrid approaches combine both direct and indirect analysis of the driver’s state. It is noteworthy that in the past, driver state analysis systems were predominantly based on indirect methods, but there has been a gradual shift towards direct and hybrid-based approaches. Table 6 presents the experimental results of DMS types from the research conducted by Baccour et al. It is evident that DMS configured with a direct approach tends to outperform those built using an indirect approach. Furthermore, it is anticipated that constructing DMS using a hybrid approach could yield even better performance than the proposed method. In this context, this paper is structured as a Direct type DMS, and it is important to note that there is room for performance improvement if a Hybrid type approach is adopted.

**Figure 13:**
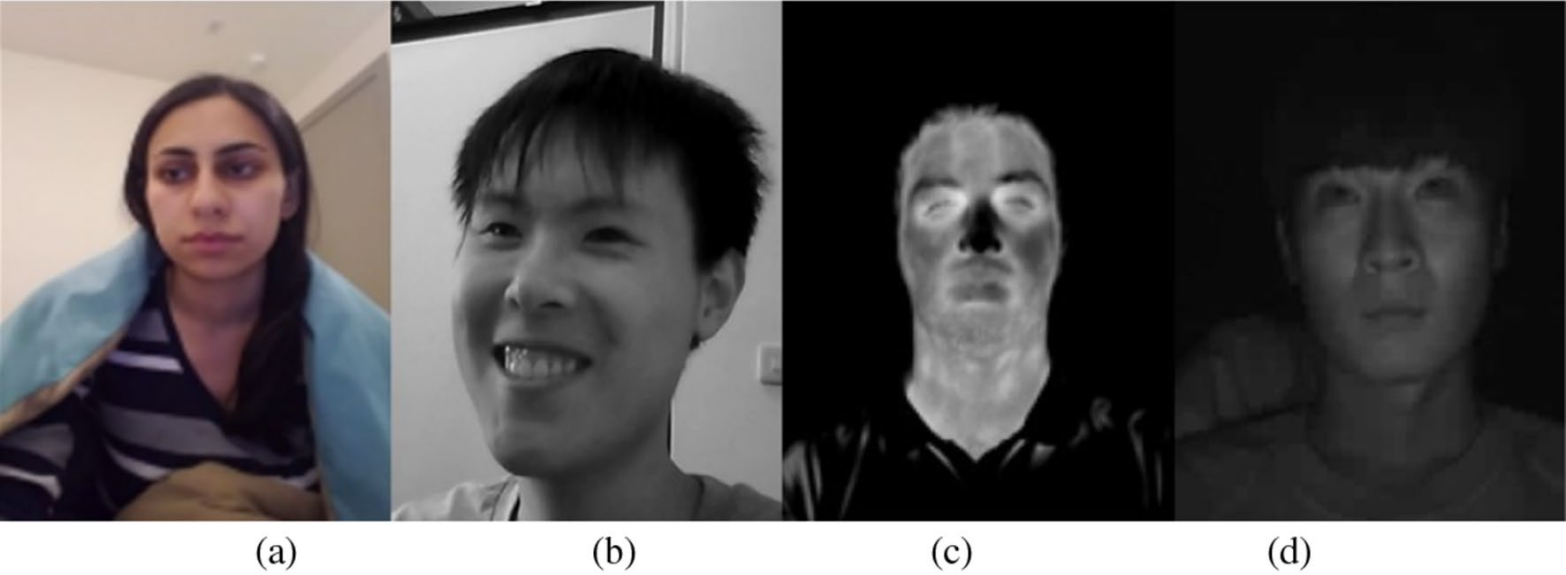
Data samples in DMS: (**a**) UTA-RLDD, (**b**) NUHU-DDD, (**c**) NIR^50^, and (**d**) ours.

In the domain of driver state analysis systems, research primarily revolves around the development of a single integrated system, in contrast to fundamental technological research such as object recognition or image enhancement. Therefore, research in this field utilizes a variety of methods and sensors. Recent DMS studies, as evident in Table 5, employ a diverse range of sensors including RGB, Depth, IR, NIR, EEG, and Motion capture for data acquisition. In this paper, to facilitate the actual commercial deployment of DMS, we custom-designed a camera tailored for driver state analysis. The IR camera from CANLAB was purposefully engineered to establish a robust DMS capable of withstanding changes in lighting conditions, offering the advantage of consistent image characteristics across varying environmental settings. Figure 13 shows examples of publicly available datasets, NIR, and sample IR datasets from this paper.

In the case of RGB cameras, denoted as Fig. 13a and b, a notable disadvantage is their susceptibility to changes in background and lighting conditions. This vulnerability is a concern, especially when aiming for practical commercial deployment of DMS. This is primarily attributed to the fact that learning-based methods, which are commonly used in DMS, can be significantly affected by changes in lighting conditions and backgrounds. Consequently, it is anticipated that such conditions may lead to a considerable amount of misclassification. On the other hand, as depicted in Fig. 13c, NIR cameras offer the advantage of analyzing various facial features, including heart rate, based on facial heat. However, they come with the disadvantage of a higher cost and variations in features depending on ambient temperature. Additionally, unlike RGB images, NIR cameras have certain limitations, including the loss of detailed facial features. While there is a growing trend of using various sensors to construct DMS to overcome these limitations, for image-based Direct driver state analysis, the IR camera utilized in this paper proves to be a suitable choice. To prevent misclassifications in learning-based approaches, it is crucial to establish an environment that is well-suited for driver detection, emphasizing the importance of creating optimal conditions for accurate DMS performance.

The IR camera employed in this paper has undergone various tests to facilitate ease of driver state analysis. Furthermore, the proposed DMS in this paper has been tested in a real embedded environment, demonstrating its capability for real-time driver state analysis. It has been proven to enable real-time driver analysis even in the Xavior environment, and with visualization and detailed optimization, it is expected to further improve its speed. While direct speed comparisons with existing DMS systems may be challenging, compared to previous papers utilizing computer vision-based technology, it is anticipated to be the most cost-effective option. Additionally, the proposed DMS uses deep learning only for driver face detection and employs CPU for tasks such as blink detection and head pose estimation, resulting in minimal GPU load. In conclusion, although DMS research has explored various sensors and data, practical commercial deployment necessitates the use of optimized algorithms and sensors tailored for driver state analysis. The DMS method and IR camera proposed in this paper offer an efficient solution for driver state analysis.

## Conclusion

The field of driver-state analysis is currently gaining significant attention due to the advancements in autonomous driving technology. In this paper, we present an innovative algorithm that analyzes the driver’s state using an infrared (IR) camera installed in the vehicle. The proposed algorithm leverages the advantages of IR cameras, which are robust to changes in lighting, making them ideal for analyzing the driver’s state in various environments, including tunnels and nighttime driving.

To efficiently analyze the driver’s face, the IR camera is strategically positioned on the steering wheel to ensure accurate monitoring. The analysis process begins with real-time face detection using YOLO V7 on the logged IR Images. Subsequently, facial landmarks are extracted from the detected face’s bounding box. These landmarks are then utilized for head pose estimation using the solvePnP algorithm and for detecting eye closure based on the eyes’ positions. To recognize inattentive situations, we employ head pose estimation to estimate the direction of the driver’s face. A method for recognizing inattentive situations based on the estimated direction is proposed. On the other hand, for drowsy driving detection, eye closure plays a vital role. We use eye closure detection filters to identify situations when the eyes are closed, and we propose a method for recognizing drowsy behavior by detecting sustained eye closure.

While numerous methods exist for driver state analysis systems, our paper focuses on an optimized approach that can be effectively incorporated into a vehicle. We design the proposed system by dividing it into two modules for efficient behavioral recognition. By integrating algorithms with fast processing speed, we introduce a driver state analysis system capable of real-time analysis. The proposed system’s efficacy was validated through testing on an in-house dataset, demonstrating excellent results. Additionally, the software information from our proposed system can be integrated with the vehicle’s hardware information to develop a complete Driver Monitoring System (DMS) in the future.

In conclusion, our proposed algorithm showcases a promising solution for driver-state analysis, offering real-time analysis capabilities and excellent performance. By integrating this system into vehicles, we can further enhance driving safety and pave the way for comprehensive DMS implementations. In the future research, we intend to merge the techniques discussed in this paper to study driver behavior through On-Board Diagnostics II (OBD-II) data, specifically focusing on steering wheel input and driving patterns. This integration will further refine our driver state assessment. Furthermore, we’re looking to tailor the proposed system for seamless operation on compact embedded boards. Our ultimate goal with the system presented in this paper is commercial deployment, and we are dedicated to rigorous testing and exploration to pave the way for its market entry.

### Supplementary Information

Supplementary Information.

## Data Availability

The raw data supporting the findings of this study are available in the supplementary material of this article. Additionally, the datasets generated and analyzed during the current study are available in the IR-Camera Datasets Repository (https://github.com/kdh6126/IR-Carmera-Datasets/). The video recordings for analysis are included in the videos.zip file within the repository. Metadata related to the videos can be found in the GT.csv, and GT_definition .csv files.
